# Relaxation capacity of cartilage is a critical factor in rate- and integrity-dependent fracture

**DOI:** 10.1038/s41598-021-88942-w

**Published:** 2021-05-04

**Authors:** G. Han, U. Chowdhury, M. Eriten, C. R. Henak

**Affiliations:** 1 Department of Mechanical Engineering, University of Minnesota, 111 Church St SE, Minneapolis, MN 55455 USA; 2 Department of Mechanical Engineering, University of Wisconsin-Madison, 1513 University Ave., Madison, WI 53706 USA; 3 Department of Biomedical Engineering, University of Wisconsin-Madison, 1550 University Ave., Madison, WI 53706 USA; 4 Department of Orthopedics and Rehabilitation, University of Wisconsin-Madison, 1111 Highland Ave., Madison, WI 53705 USA

**Keywords:** Mechanical engineering, Musculoskeletal system

## Abstract

Articular cartilage heals poorly but experiences mechanically induced damage across a broad range of loading rates and matrix integrity. Because loading rates and matrix integrity affect cartilage mechanical responses due to poroviscoelastic relaxation mechanisms, their effects on cartilage failure are important for assessing and preventing failure. This paper investigated rate- and integrity-dependent crack nucleation in cartilage from pre- to post-relaxation timescales. Rate-dependent crack nucleation and relaxation responses were obtained as a function of matrix integrity through microindentation. Total work for crack nucleation increased with decreased matrix integrity, and with decreased loading rates. Critical energy release rate of intact cartilage was estimated as 2.39 ± 1.39 to 2.48 ± 1.26 kJ m^−2^ in a pre-relaxation timescale. These findings showed that crack nucleation is delayed when cartilage can accommodate localized loading through poroviscoelastic relaxation mechanisms before fracture at a given loading rate and integrity state.

## Introduction

Articular cartilage is a dissipative and heterogeneous thin layer protecting the ends of bones in diarthrodial joints. Cartilage is composed of a tightly woven solid matrix hydrated by fluid. The solid matrix mainly consists of a collagen network (about 15–22% of wet weight^1^) intertwined with proteoglycans (PGs) with sulfated glycosaminoglycan (GAG) side chains (about 4–7% of wet weight^1^). Negatively charged GAGs generate swelling force via osmotic pressure and electrostatic repulsion between charges and thus provide compressive strength to cartilage^1,2^. The collagen network is under tension, counterbalancing the GAG-induced swelling force^3,4^. The cohesive strength of cartilage mainly stems from the collagen network^5,6^.

**Figure 1:**
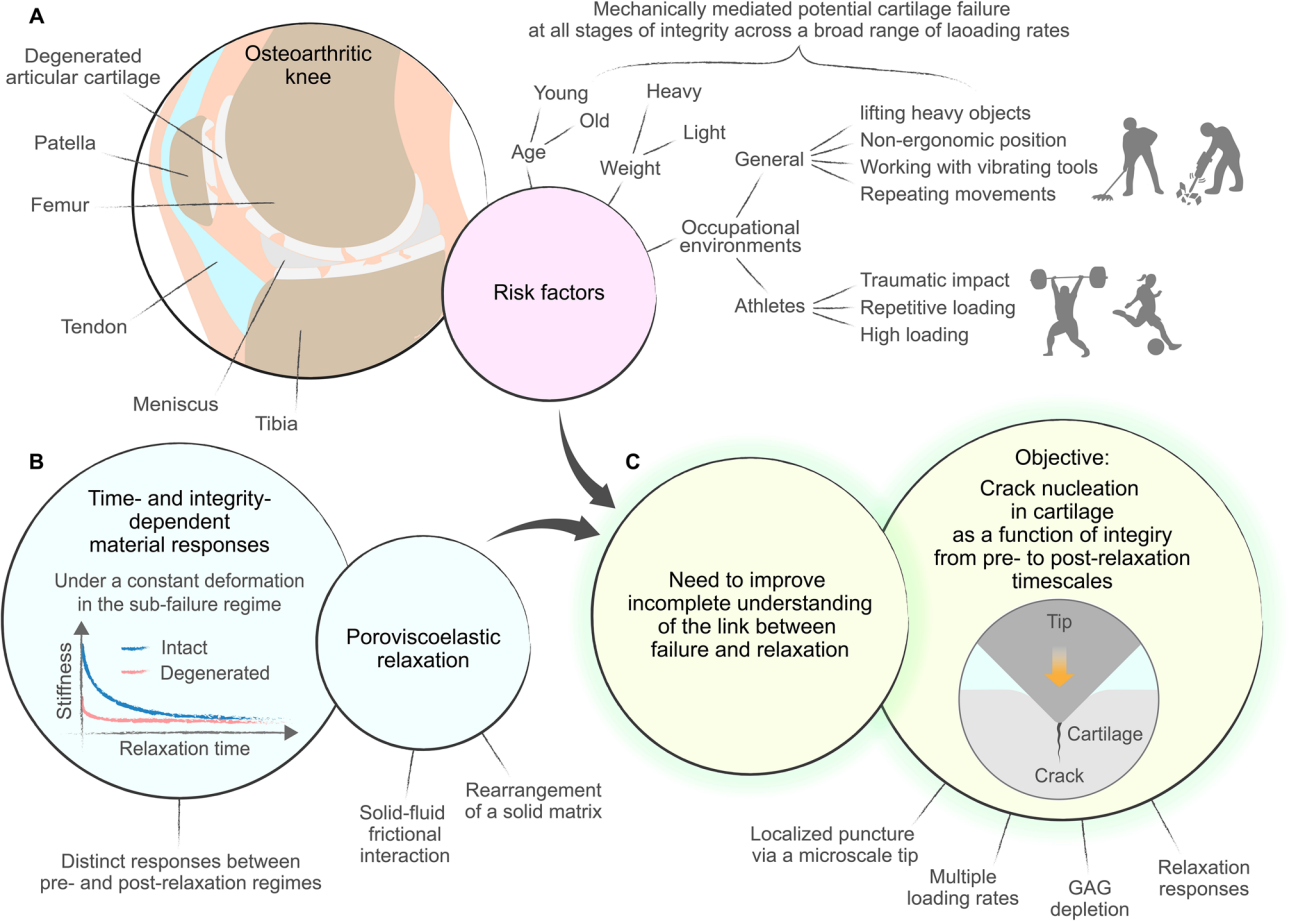
Schematic diagram of a strategic research plan. (**A**) Risk factors for osteoarthritis. (**B**) Time- and integrity-dependent cartilage responses observed in a sub-failure regime. (**C**) Objective of the current study. The link between cartilage failure and relaxation is understood by investigating crack nucleation in cartilage as a function of integrity from pre- to post-relaxation time scales.

Articular cartilage exhibits impressive resistance to failure under a range of in vivo activities but is degenerated through osteoarthritis (OA), which results from mechanically mediated damage (Fig. 1A). OA is both highly prevalent with increasing incidence (around 300 million people globally)^7–9^. Although OA can occur at any age, it is more prevalent in older adults^10^. Risk factors for OA include a variety of situations that increase joint loading, including lifting heavy objects, obesity, working in vibrating vehicles or with vibrating tools, repeating the same movements, and working at a pace set by a machine^11,12^. Representative occupations at risk are cleaners, masons, construction workers, and agricultural workers^12^. In addition, athletes are at relatively high risk of OA due to repetitive or high traumatic impact and loading on joints^13,14^. Ice hockey, soccer, wrestling, weight lifting, and boxing all show relatively high OA^15^. Unfortunately, OA is a degenerative and progressive disease with unlikely recovery of damaged areas^16,17^. Current treatments are mainly focused on symptom control, and the only treatment for end-stage OA is joint replacement^16,17^. Therefore, it is important to understand cartilage failure mechanisms under various exposure conditions such as external loading and cartilage matrix integrity to delay the onset and progression of OA (Fig. 1A).

Cartilage exhibits poroviscoelastic (PVE) relaxation mechanisms. Poroelastic (PE) relaxation results from drag generated by pressure-induced fluid flow in pore space and dissipates energy through solid–fluid frictional interactions^18–20^ (Fig. 1B). Intrinsic viscoelastic (VE) relaxation occurs due to the molecular rearrangement of a dense solid matrix^18,21,22^. PVE relaxation mechanisms give rise to rate-dependent dissipative and mechanical responses of cartilage^19,21–23^ and thus are essential elements for understanding cartilage behavior under external loading from a range of in vivo activities. In this study, the pre-relaxation regime is when the loading timescales are shorter than cartilage relaxation timescales, and the post-relaxation regime is when the loading timescales are longer than cartilage relaxation timescales. Relaxation times are impacted by loading conditions and solid matrix integrity.

In the sub-failure regime, dissipative and mechanical cartilage responses have been investigated across a broad range of loading rates as a function of solid matrix integrity (Fig. 1B). Previous studies in the sub-failure regime showed that PVE relaxation governs rate-dependent cartilage responses from pre- to post-relaxation timescales and depends on the degree of solid matrix integrity^19,22–26^. For instance, cartilage’s resistance to deformation was much higher when a loading timescale was shorter than a PVE relaxation timescale^24,27^. Uncoupled cartilage dissipation mechanisms at multiple length scales (5–100 Hz) showed that intrinsic VE dissipation governed the basis of sustained dissipation, and PE dissipation additionally increased total dissipation at a small contact length^23^. Dynamic responses of intact and GAG-depleted cartilage (1 Hz–10 kHz covering walking to impact loading) showed that GAG depletion shifted the peak of PE dissipation to a high frequency as a result of an increased pore size for fluid flow and decreased cartilage resistance to being deformed^28^. GAG-depleted cartilage simulated matrix integrity in the early stages of OA^28^. These previous studies suggested that PVE relaxation can provide new insights into understanding mechanically mediated cartilage failure under loading regimes relevant to in vivo activities, spanning from pre- to post relaxation timescales.

In contrast to the sub-failure regime, little is known about the influence of loading rates and tissue’s PVE relaxation and solid matrix integrity on cartilage failure (Fig. 1C). Prior efforts to comprehend load-induced cartilage failure showed rate-dependent failure characteristics. For example, cartilage failure slowed down with necking phenomena under relatively low-frequency tensile loading^29^. Large crack lengths and severe damage to microstructural features were induced under relatively high-frequency compressive loading^30,31^. Recently, the authors and others found that impact loading, simulating the onset of post-traumatic OA, caused fissures and cracks^32–34^ and that crack nucleation in cartilage was largely rate-dependent^35,36^. In particular, the authors observed that the solid matrix around the crack surfaces underwent larger relaxation and rearrangement at the slow loading rate compared to the fast loading rate^36^. Tensile creep and uniaxial tension tests at a slow loading rate showed that PG extraction expedited the reorganization and alignment of the collagen network but did not influence the failure strength of cartilage^6^. Although these previous studies showed that cartilage failure is rate-dependent and is associated with PVE relaxation, a possible link between rate-dependent cartilage failure and PVE relaxation was not clearly made. It is difficult to find the link by combining previous studies because failure was not investigated from pre- to post-relaxation timescales and PVE relaxation corresponding to the failure was not examined. Furthermore, the effect of solid matrix integrity on rate-dependent cartilage failure is currently missing in the literature. To summarize, mechanically mediated cartilage failure can be hypothesized to depend on loading rates and solid matrix integrity^37,38^. The current study tests this hypothesis by investigating crack nucleation in cartilage as a function of solid matrix integrity across pre- to post-relaxation time scales (Fig. 1C).

In this study, crack nucleation in intact and GAG-depleted cartilage was induced across a broad range of loading rates through microindentation. GAG-depleted cartilage simulated the early stages of OA^39,40^. Microindentation with a sharp axisymmetric probe enabled localized crack nucleation with smaller standard deviations in comparison to macroscopic testing^36,41^. Crack nucleation was identified as a sudden drop in load and was characterized by the critical load, displacement, and total work at onset of failure. In addition, PVE relaxation responses of intact and GAG-depleted cartilage were measured with the same testing system used for crack nucleation. Finally, crack nucleation results combined with the relaxation responses provided total work required for crack nucleation in intact and GAG-depleted cartilage across pre- to post-relaxation timescales. Relaxation-dependent crack surfaces were visualized via an optical microscope. These experimental results demonstrated that crack nucleation in intact and GAG-depleted cartilage was governed by how quickly cartilage was able to accommodate localized loading through PVE relaxations.

## Results

### Crack nucleation depends on solid matrix integrity and loading rates

**Figure 2:**
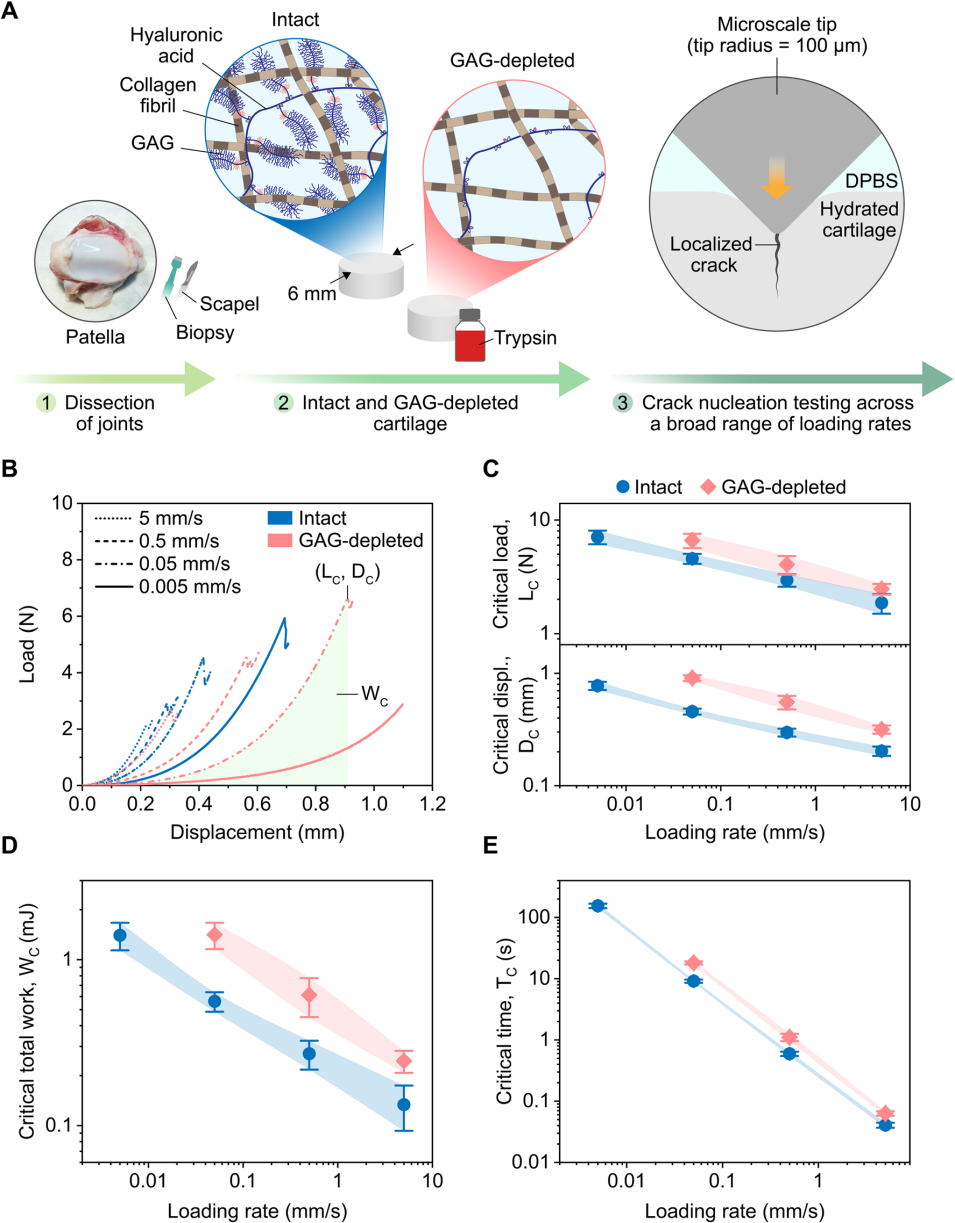
Crack nucleation in cartilage as a function of integrity across a broad range of loading rates. (**A**) Schematic illustrations of crack nucleation testing on intact and GAG-depleted cartilage via a microscale tip. (**B**) Representative load–displacement curves. Crack nucleation curves were analyzed with critical parameters: (**C**) critical load, critical displacement, (**D**) critical total work, and (**E**) critical time. The schematic diagrams are not to scale. Averages and standard deviations (connected with the modified Bézier curves) were reported (n = 10).

The effects of loading rates on cartilage failure as a function of integrity were investigated by inducing crack nucleation in intact and GAG-depleted cartilage with a microscale tip at multiple loading rates (0.005–5 mm s^−1^) (Fig. 2A). The moment of crack nucleation was identified through a sudden drop in the load response and was quantified with critical parameters (Fig. 2): critical load, *L*_*C*_, critical displacement, *D*_*C*_, critical total work, *W*_*C*_, and critical time, *T*_*C*_. All of the loading conditions except for GAG-depleted cartilage at 0.005 mm s^−1^ achieved crack nucleation. Loading rates and GAG depletion significantly affected critical parameters (Fig. 2B). For all loading rates, the recorded loads were higher for intact cartilage than those for GAG-depleted cartilage (Fig. 2B and Fig. S1A) due to GAG-depletion induced loss of compressive stiffness. GAG depletion approximately increased critical loads by 30–45% at matching loading rates (5–0.05 mm s^−1^: *p* < 0.01). For both intact and GAG-depleted tissues, critical loads at the onset of crack nucleation decreased as the loading rates increased (Intact and GAG-depleted: *p* < 0.0001) (Fig. 2C). Critical load of intact cartilage at 0.005 mm s^−1^ (7.08 ± 0.97 N) was approximately 4 times higher on average than that at 5 mm s^−1^ (1.86 ± 0.37 N). Critical load of GAG-depleted cartilage at 0.05 mm s^−1^ (6.61 ± 0.96 N) was approximately 3 times higher on average than that at 5 mm s^−1^ (2.46 ± 0.27 N). GAG depletion increased critical displacement by approximately 55–100% at corresponding loading rates (5–0.05 mm s^−1^: *p* < 0.001). Critical displacements of intact and GAG-depleted cartilage decreased with increasing loading rates (intact and GAG-depleted: *p* < 0.0001) (Fig. 2C). Critical displacement of intact cartilage at 0.005 mm s^−1^ (0.78 ± 0.07 mm) was nearly 4 times larger on average than that at 5 mm s^−1^ (0.20 ± 0.02 mm). Critical displacement of GAG-depleted cartilage decreased nearly 3 times on average from 0.05 mm s^−1^ (0.91 ± 0.06 mm) to 5 mm s^−1^ (0.32 ± 0.03 mm). GAG depletion increased total work required for crack nucleation by approximately 80–150% across all of the loading rates (5–0.05 mm s^−1^: *p* < 0.001). For both intact and GAG-depleted cartilage, critical total work sharply decreased as loading rates increased (intact and GAG-depleted: *p* < 0.0001) (Fig. 2D). Critical work of intact cartilage at 0.005 mm s^−1^ (1.41 ± 0.27 mJ) was nearly 11 times larger on average than that at 5 mm s^−1^ (0.13 ± 0.04 mJ). Critical work of GAG-depleted cartilage went down nearly 6 times from 0.05 mm s^−1^ (1.42 ± 0.26 mJ) to 5 mm s^−1^ (0.25 ± 0.04 mJ). GAG depletion delayed critical time by approximately 55–100% across all of the loading rates (5–0.05 mm s^−1^: *p* < 0.001). Critical times of intact and GAG-depleted cartilage decreased with increasing loading rates (intact and GAG-depleted: *p* < 0.0001) (Fig. 2E). Critical time of intact cartilage at 0.005 mm s^−1^ (155.17 ± 13.04 s) was around 3900 times larger on average than that at 5 mm s^−1^ (0.04 ± 0.004 s). Critical time of GAG-depleted cartilage increased around 300 times from 5 mm s^−1^ (0.06 ± 0.01 s) to 0.05 mm s^−1^ (18.16 ± 1.09 s).

### GAG-depleted cartilage relaxes faster than intact cartilage

**Figure 3:**
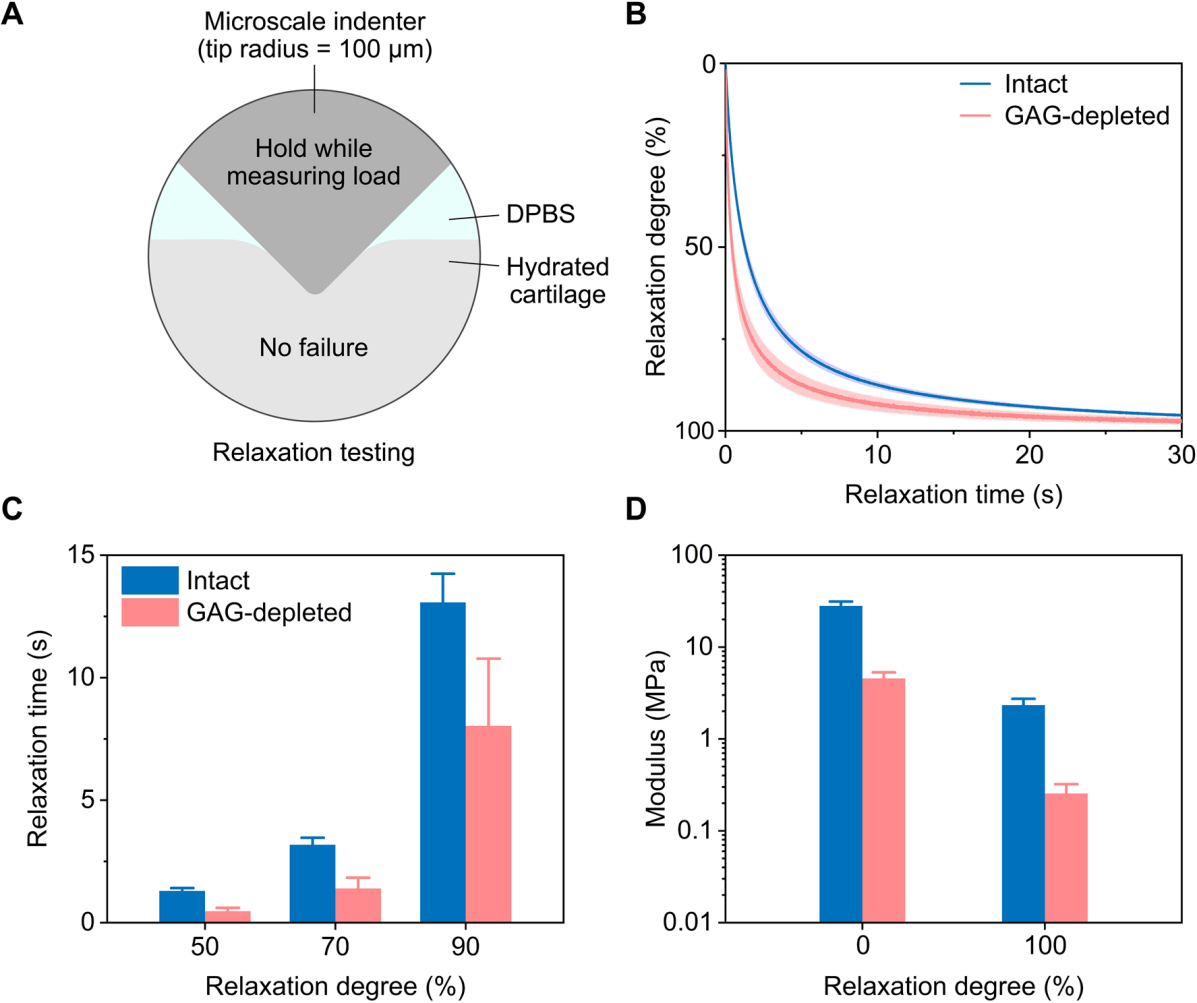
Relaxation responses of cartilage as a function of integrity. (**A**) Schematic illustrations of relaxation testing on intact and GAG-depleted cartilage via a microscale tip. (**B**) Relaxation curves as a function of time. (**C**) Relaxation times at different relaxation degrees. (**D**) Elastic moduli at two extreme relaxation degrees. The schematic diagram is not to scale. Averages and standard deviations were reported (n = 10).

Integrity-dependent cartilage relaxation was examined by measuring relaxation responses of intact and GAG-depleted cartilage via a microscale tip at a constant displacement (Fig. 3A). The relaxation response of GAG-depleted cartilage was distinct from that of intact cartilage (Fig. 3B). The peak load of intact cartilage (2.12 ± 0.26 N) at an unrelaxed state (after 0 s of relaxation) was nearly 6.5 times higher on average than that of GAG-depleted cartilage (0.33 ± 0.05 N) (Fig. 3B and Fig. S1B). The equilibrium load of intact cartilage (0.18 ± 0.03 N) at a relaxed state (after 200 s of relaxation) was 9 times higher on average than that of GAG-depleted cartilage (0.02 ± 0.005 N) (Fig. 3B and Fig. S1B). GAG-depleted cartilage showed much faster relaxation than intact cartilage (50–90% of the total relaxation: *p* < 0.01) (Fig. 3B,C). Relaxation times were determined as time required to reach a certain percentage of the total relaxation based on a normalized experimental relaxation curve (Fig. 3B,C). For intact cartilage, relaxation times at 50, 70, and 90% of the total relaxation were 1.29 ± 0.12 s, 3.18 ± 0.29 s, and 13.07 ± 1.17 s, respectively (*p* < 0.0001). For GAG-depleted cartilage, relaxation times at 50, 70, and 90% of the total relaxation corresponded to 0.47 ± 0.14 s, 1.40 ± 0.44 s, and 8.04 ± 2.74 s, respectively (*p* < 0.0001). Intact cartilage showed higher elastic moduli compared to GAG-depleted cartilage (unrelaxed and relaxed: *p* < 0.001) (Fig. 3D and Fig. S1B). Elastic moduli of intact cartilage at unrelaxed and relaxed states were 28.06 ± 3.23 MPa and 2.33 ± 0.40 MPa, respectively. Elastic moduli of GAG-depleted cartilage at unrelaxed and relaxed states were 4.56 ± 0.74 MPa and 0.25 ± 0.07 MPa, respectively. Some discrepancies in unrelaxed (instantaneous) and relaxed (equilibrium) moduli of intact and GAG-depleted cartilage between the current and previous studies could originate from age, species, residual GAG content, and testing conditions such as loading rates and load-associated contact lengths^24,28,42^.

### Relaxation as a function of solid matrix integrity governs crack nucleation behavior

**Figure 4:**
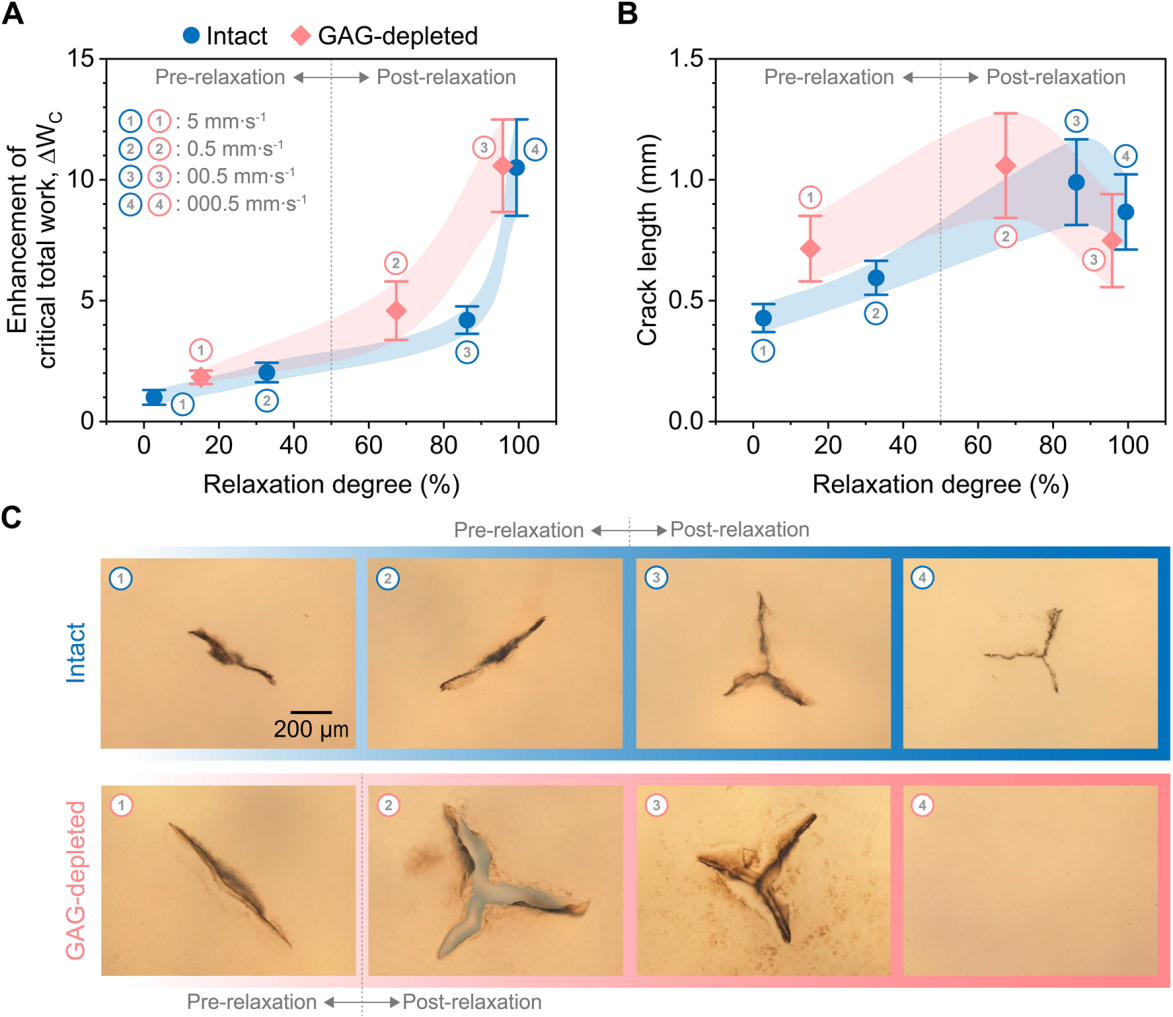
Relaxation-dependent crack nucleation in cartilage as a function of integrity. (**A**) Enhancement of critical total work with greater relaxation degrees. The enhancement of critical total work was obtained by dividing the data points by the minimum critical total work of intact cartilage at 5 mm s^−1^. (**B**) Crack lengths. (**C**) Brightfield images of cracks. The numbers 1, 2, 3, and 4 correspond to the loading rates of 5 mm s^−1^, 0.5 mm s^−1^, 0.05 mm s^−1^, and 0.005 mm s^−1^, respectively. Averages and standard deviations (connected with the modified Bézier curves) were reported (n = 10).

A relationship between crack nucleation in intact and GAG-depleted cartilage and PVE relaxation degree was investigated by linking critical time for crack nucleation with relaxation time (Fig. 4A). For intact cartilage, critical time at 5 mm s^−1^ and 0.5 mm s^−1^ (Fig. 2E) was shorter than 50% relaxation time (Fig. 3C), and thus crack nucleation was induced in a pre-relaxation timescale. Conversely, critical time at 0.05 mm s^−1^ and 0.005 mm s^−1^ in intact cartilage belonged to a post-relaxation timescale. For GAG-depleted cartilage, critical time at 5 mm s^−1^ was shorter than 50% relaxation time, and therefore crack nucleation was generated in a pre-relaxation timescale. Critical time at 0.5 mm s^−1^ and 0.05 mm s^−1^ was within a post-relaxation timescale for GAG-depleted cartilage. For both intact and GAG-depleted cartilage, much less critical total work was needed to induce crack nucleation in a pre-relaxation timescale compared to a post-relaxation time scale. Crack nucleation in GAG-depleted cartilage occurred at much higher relaxation degrees and critical total work than in intact cartilage. Although the critical total work curves (Fig. 4A) visualized an important role of PVE relaxation degree in crack nucleation, there were sources for possible shifts of the curves. The loading ramp rate before the relaxation period (Fig. 3B) was not instantaneous loading to prevent the onset of crack nucleation during the ramp loading. Analyzing the average relaxation time with a previous study^43^ suggested that the relatively slow loading ramp rate could shift the curves for intact and GAG-depleted tissues toward higher relaxation degrees by about 1% and about 3%, respectively. In addition, the dependence of bulk PVE relaxation responses on a contact radius between the indenter and tissue can cause the shifts of the curves. This dependence results from PE relaxation because it depends on the square of a contract radius, controlling the PE diffusion length^19,44^. The comparison between the current study at a microscale length with our previous study at a macroscopic length showed that although the ratio of the square of contact radii at the two length scales was about 66, the ratio of average relaxation times between the two studies was only about 5 for intact and about 1 for GAG-depleted cartilage, respectively. Based on this comparison, as the ratio of a contact radius during the relaxation measurement to a contact radius experienced during crack nucleation tests was small (intact: < about 6 and GAG-depleted: < about 8), the effect of indentation depth on relaxation times would be minor.

### Cracks become longer and more branched with greater relaxation degrees

A transition in crack shapes from pre- to post-relaxation timescales was investigated via optical images of cracks stained with India ink (Fig. 4B,C). Crack lengths of intact cartilage increased from 430 ± 60 µm in a pre-relaxation timescale (5 mm s^−1^) to 865 ± 155 µm in a post-relaxation timescale (0.005 mm s^−1^) (*p* < 0.0001) (Fig. 4B). The maximum crack length for intact cartilage of 990 ± 175 µm was observed at 0.05 mm s^−1^. The number of branches extended from the center of cracks increased with increasing loading rates from 2 in a pre-relaxation timescale (5 mm s^−1^) to 3 in a post-relaxation timescale (0.005 mm s^−1^) (*p* < 0.0001) (Fig. 4C and Fig. S2A). Similarly, crack lengths of GAG-depleted cartilage were rate-dependent (*p* < 0.01) and slightly increased from 715 ± 135 µm in a pre-relaxation timescale (5 mm s^−1^) to 750 ± 190 µm in a post-relaxation timescale (0.05 mm s^−1^) (Fig. 4B). The maximum crack length for GAG-depleted cartilage was 1060 ± 215 µm at 0.5 mm s^−1^. The crack lengths (715 ± 135 µm) of GAG-depleted cartilage in a pre-relaxation timescale may be overestimated because possible sequential crack propagation was observed after the major crack nucleation. However, we were able to induce a single major crack nucleation event for intact and GAG-depleted cartilage at other loading rates without sequential crack events. The number of branches increased from two in a pre-relaxation timescale (5 mm s^−1^) to three in a post-relaxation timescale (0.05 mm s^−1^) (*p* < 0.0001) (Fig. 4C and Fig. S2B). For GAG-depleted cartilage loaded at 0.005 mm s^−1^, no evidence of crack nucleation was observed; i.e., no sudden load drops in microindentation response and no crack opening observed after staining.

## Discussion

### Poroviscoelastic relaxation at slow loading rates delays cartilage fracture

PVE relaxation mechanisms play a crucial role in determining the onset of cartilage failure, supported by critical total work combined with crack images showing that crack nucleation in intact and GAG-depleted cartilage was relaxation-dependent. When crack nucleation in intact and GAG-depleted cartilage occurred in a post-relaxation timescale (Fig. 4A) where critical time (Fig. 2E) was larger than 50% relaxation time (Fig. 3B,C), critical total work was nearly an order of magnitude higher than that in a pre-relaxation timescale. This was because cartilage had sufficient time to accommodate localized microindentation strains and thus distribute stresses over larger tissue volumes. This stress-diffusion process, as quantified by the degree of PVE relaxation, led to delays in crack nucleation^36^. Besides, a substantial portion of applied mechanical work was consumed by the PVE relaxation; specifically by the solid matrix rearrangement and solid–fluid frictional interactions. In contrast, microindentation over a pre-relaxation timescale led to localized stress and strains in the vicinity of the probe and thus provided strain energy with minimal losses to be used directly for crack nucleation. As a result, critical total work in a pre-relaxation timescale was much smaller. A transition in crack branches from pre- to post-relaxation timescales reiterated that PVE relaxation played a crucial role in crack nucleation in intact and GAG-depleted cartilage (Fig. 4B,C). In addition, the finding about critical total work across a broad range of relaxation degree (Fig. 4A) showed that critical total work was more sensitive to relaxation degree in a post-relaxation timescale. This could be because kinematic fibril rearrangement actively occurred in a post-relaxation timescale (intact: $\ge$ 85% relaxation degree and GAG-depleted: $\ge$ 65% relaxation degree) with the large exudation of fluid in the vicinity of the tip, resulting in the compaction of the tissue. These findings suggested that physical activities occurring over timescales shorter than relaxation time constants of the tissue would make cartilage significantly more vulnerable to failure.

### Accelerated relaxation capacity delays cartilage fracture at given loading rates

GAG depletion allowed for access to the effects of PVE relaxation timescales on crack nucleation, reiterating that the relaxation degree governed crack nucleation even at different degrees of solid matrix integrity. GAG depletion accelerated cartilage PVE relaxation (Fig. 3B,C). This could be due to accelerated PE relaxation, resulting from the increased pore size of cartilage, or from possible alteration in time constants of intrinsic VE relaxation, resulting from altered nonfibrillar matrix density and collagen matrix configuration^6,24,28,42^. Crack nucleation in GAG-depleted cartilage required higher critical total work and relaxation degree than intact cartilage at corresponding loading rates (Fig. 4A). It has been reported that the collagen network in tension dominantly governed cohesive strength of cartilage rather than PGs with GAG side chains^5,6,45^. Consequently, the current findings, combined with the previous studies, indicated that accelerated PVE relaxation after GAG depletion delayed an increase in stress underneath the microscale tip^36^ by rapidly accommodating localized loading at given loading rates. This rapid accommodation of GAG-depleted cartilage by accelerated PVE relaxation was likely to originate from the enhanced freedom of collagen fibril rearrangement in the absence of confined non-fibrillar matrixes, resulting in increased critical total work, critical load, and critical displacement for crack nucleation.

### Estimations of critical energy release rates from crack morphologies in the pre-relaxation timescale

In the pre-relaxation timescale, GAG-depleted cartilage was likely to require less energy per unit fracture surface area than intact cartilage. The consistent line shapes of cracks in intact and GAG-depleted cartilage in pre-relaxation timescales (Fig. 4C) allowed for quick estimates of critical energy release rates via the energy-balance model of sharp-tipped punch penetration^46,47^. Detailed information about the model is given in the supplementary text and Fig. S3. The estimated critical energy release rates of intact cartilage were 2.39 ± 1.39 to 2.48 ± 1.26 kJ m^−2^ (5–0.5 mm s^−1^) and were higher than the upper bound of previously reported values for cartilage (0.14 ± 0.08–1.46 ± 0.91 kJ m^−2^ ^48^, 0.83 ± 0.19–1.12 ± 0.2 kJ m^−2^ ^41^, and 0.8 ± 1.05–1.17 ± 1.13 kJ m^−2^ ^49^). The discrepancies could be due to species, age, and testing configuration such as frictional interactions between probe and samples^50^. The estimated critical energy release rate of GAG-depleted cartilage was 2.09 ± 2.99 kJ m^−2^ (5 mm s^−1^). However, this average value might have been overstated due to one data point of 10.43 kJ m^−2^. This was because the crack length (408.10 µm) was smaller than other cases (749.30 ± 86.53 µm), consistent with a relatively small drop in a load response. The average value without the one data point was 1.17 ± 0.62 kJ m^−2^ (5 mm s^−1^).

### Relaxation-dependent crack branching provides insight into cartilage failure

Relaxation degree before crack nucleation affected crack lengths and morphologies. Crack lengths increased and more crack branching occurred from pre- to post-relaxation timescales (Fig. 4B,C and S2). Our observations as well as the literature suggested that cracks should nucleate at a single-line cut due to stress intensities. Subsequent release of energy during propagation of cracks could cause instabilities and fragmentation, especially when single-line cracks cannot increase free surfaces to balance large strain energies to be released. This mismatch can occur in ballistic loading cases as well as in quasistatic perforation of thin shells and plates^51,52^. Dynamic crack propagation and branching cannot be observed in our test setup. Besides, even though we varied loading rates to 5 mm s^−1^, those speeds are still orders of magnitude lower than shear wave speeds, *C*_S_, of the tissue (*C*_S_ = $\sqrt {G/\rho }$ ~ 1–10 m/s where *G*: shear modulus and $\rho$: density of the medium). Thus, a quasistatic loading scenario was more applicable to our microindentation-based crack nucleation tests. For slow loading cases studied (a post-relaxation timescale), the sphero-conical indenter penetrated significantly larger depths than fast loading cases (a pre-relaxation timescale) (Fig. 2C). At those depths, significant tensile strains accumulated due to the stretching of collagen fibers around the sharp tip (see the model in the supplementary material). This kinematic stretching combined with large strain energy accumulated in a post-relaxation timescale led to the splitting of the single-line cut in a three-branch crack as shown in Fig. 4C. Note that kinematic alignments and stretching of collagen fibers were limited in a pre-relaxation timescale (see SEM images in our previous work^36^). Therefore, single-line cuts could create sufficient crack area to balance corresponding energy releases. To the best of the authors’ knowledge, the current study is the first to report crack branching in cartilage. Despite being beyond the scope of this study, understanding the factors driving crack branching in cartilage could offer translational benefits given the tissue’s limited healing ability.

### Estimated threshold flaw sizes of cartilage provide insight into catastrophic fracture due to pre-existing cracks

**Figure 5:**
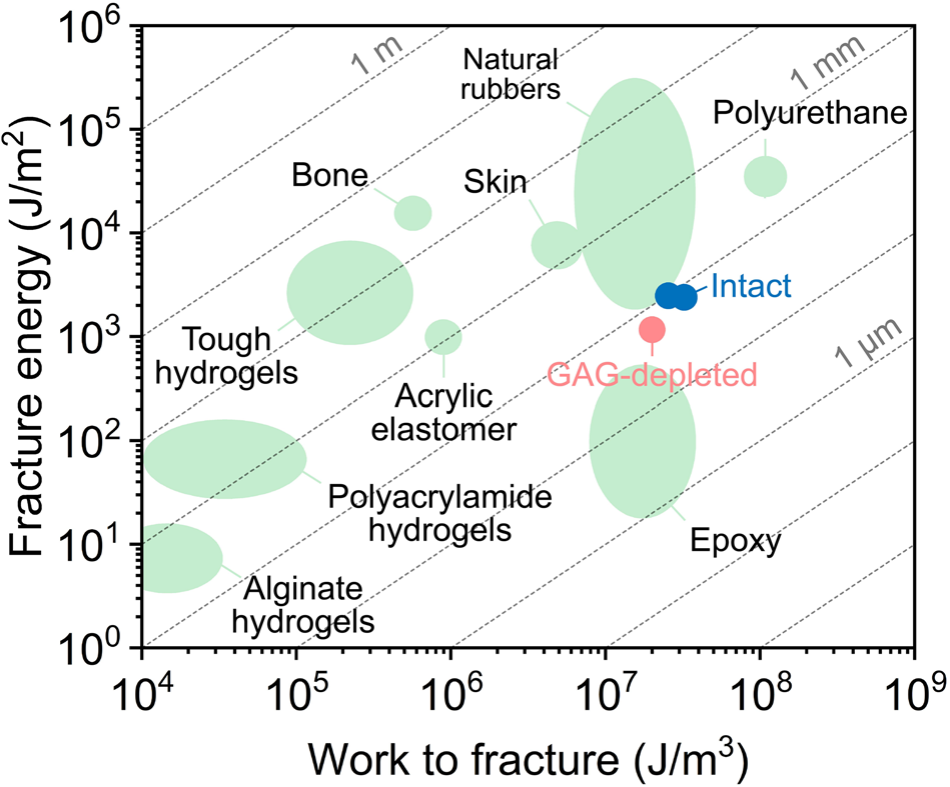
Comparison of intact and GAG-depleted cartilage with other materials in terms of the fracture energy, work to fracture, and length of flaw sensitivity. The diagonal lines represent the length of flaw sensitivity, calculated by dividing fracture energy (critical energy release rates) by work to fracture. The mechanical properties of other materials were from previous studies^53,65–69^. The conversion of the mechanical properties into this plot was explained in a previous study^53^. The data points of intact and GAG-depleted cartilage were obtained from a pre-relaxation timescale, and the detailed process was included in the supplementary text, Fig. S3 and Fig. S4.

Another measure of cartilage’s toughness is its tolerance to pre-existing cracks. Soft materials such as elastomers and double-network hydrogels exhibit excellent stretchability when pre-existing cracks have lengths below a threshold flaw size estimated as the ratio of toughness to work to rupture^53^. As noted above, the microindentation-based crack nucleation tests can readily deliver work to rupture (referred to as critical work above). However, this work is an apparent quantity affected by dissipation, strain energy density, and deformed volume. Since microindentation localizes large strains in the vicinity of the contact scaling with a contact radius, strain energy densities, *S*_*D*_, can be approximated by the ratio of the measured work to rupture, *W*_*C*_, to the cube of contact radius, *a*, (${S}_{D}\sim \frac{{W}_{C}}{{a}^{3}}$). Note that strain energy densities are non-homogenous under the indenter, and so the approximation should be treated as an averaged quantity. Besides, dissipated energy is included in that estimate, and thus, it should be treated as an upper boundary of critical strain energy densities (work to fracture). The work to fracture based on critical total work (Fig. S4) and the critical energy release rates (fracture energy) of intact and GAG-depleted cartilage in a pre-relaxation timescale (supplementary text and Fig. S3) allowed us to estimate their threshold flaw sizes (Fig. 5). These thresholds for intact cartilage at 5 mm s^−1^ and 0.5 mm s^−1^ were 78.16 $\pm$ 48.91 µm and 99.49 $\pm$ 55.53 µm, respectively. The threshold for GAG-depleted cartilage at 5 mm s^−1^ was 63.47 $\pm$ 41.32 µm with 1.17 ± 0.62 kJ m^−2^ critical energy release rate (105.55 $\pm$ 138.65 µm with 2.09 ± 2.99 kJ m^−2^). Those threshold flaw sizes are larger than cartilage’s structural features (chondrocytes: ~ 13 µm^54^, spacing between GAG chains: ~ 3 nm^55^, and spacing between collagen fibrils: ~ 100 nm^56^). Therefore structural features are unlikely to lead to a flaw-sensitive region where the tissue's toughness is compromised. However, load-induced cracks with lengths larger than those thresholds were observed in the literature^30,57^. Those pre-existing flaws can accelerate the catastrophic failure of cartilage. We did not report similar thresholds at post-relaxation timescales since the crack morphology and potential substrate effects blur estimation of critical strain energy release rates (fracture energy) and work to fracture.

### Crack nucleation results improve understanding of cartilage failure during physiological activities

The current findings about relaxation- and integrity-dependent crack nucleation can provide insight into cartilage failure under physiological activities. Bulk strain rates of intact and GAG-depleted cartilage (5 mm s^−1^) in a pre-relaxation timescale were 3.35 ± 0.25 s^−1^ and 3.29 ± 0.21 s^−1^, respectively, and fall within the lower bound of the strain rates during walking, jumping, and running^28,58,59^. Bulk strain rates of intact (0.005 mm s^−1^) and GAG-depleted (0.05 mm s^−1^) cartilage in a post-relaxation timescale were 0.0032 ± 0.0003 s^−1^ and 0.032 ± 0.029 s^−1^, respectively, and could represent human resting. Bulk strain rates were estimated by dividing the ratio of critical displacement to thickness by critical time. The interpretation of the current findings with the bulk strain rates suggested that when load-associated time during physiological activities is in a pre-relaxation timescale, cartilage failure could easily occur with relatively small total work.

## Limitations

This study filled gaps in knowledge related to the link between cartilage failure and PVE relaxations, but limitations should be explored further. The experimental conditions were different from in vivo conditions. One of the possible differences could be the pre-compression levels of cartilage by body weight^60^ at the moment of mechanically mediated crack nucleation. Since the unrelaxed and relaxed elastic moduli of GAG-depleted cartilage were lower than those of intact cartilage (Fig. 3D), the pre-compression levels of GAG-depleted cartilage would be higher compared to those of intact cartilage. However, the current findings with the fully hydrated cartilage thickness under no initial compression allowed us to compare crack nucleation in cartilage as a function of solid matrix integrity from pre- to post-relaxation timescales. The fundamental comparison could lay the foundation for understanding more complicated cases in in vivo conditions through experiments and simulations. Minor cracks in the initial stages of indentation might not be captured due to the noise floor in the load cell. Given the rigid kinematic constraint imposed by the indenter tip with a tip radius of 100 μm, these minor cracks were expected to have a crack length much smaller than the tip radius. As cartilage is a hydrated material, the identification of these minor cracks with an optical microscope is challenging. Moreover, these pre-existing flaws were smaller than the threshold flaw sizes of intact and GAG-depleted cartilage, and therefore we do not anticipate much compromise on cartilage failure responses in our measurements. In this sense, crack nucleation investigated in this study was defined as a major crack nucleation event that was detected with the current instrument. In order to distinguish between minor and major crack nucleation events, experimental instruments with higher force and displacement resolutions are required. Cartilage integrity was only controlled by depleting GAGs and non-fibrillar components via trypsin digestion, and thus the current study did not explore the effect of other fibrillar components (e.g., collagen fibrils) on relaxation-dependent cartilage failure. Cartilage relaxation behavior depends on temperature with accelerated relaxation at higher tempertures^61^, so testing at different temperatures would be expected to shift the time at which the pre- and post-relaxation timescales occur. As the indenter geometry governs stress distributions in the vicinity of the indenter tip, it can affect the numerical values of the crack nucleation results and the transition timescale from pre- to post-relaxation regimes^36,44^.

## Conclusions

In conclusion, this work examined a link between rate-dependent crack nucleation and PVE relaxation as a function of cartilage integrity. An axisymmetric micro-indenter effectively generated rate-dependent crack nucleation in intact and GAG-depleted cartilage at known locations from pre- to post-relaxation timescales. Rate-dependent crack nucleation was governed by the degree of PVE relaxation at given cartilage integrity. The degeneration of solid matrix integrity by GAG depletion allowed access to a different PVE relaxation timescale, significantly decreased relaxation time, and increased critical total work for cartilage failure. These results indicated that GAG depletion enhanced the capacity of kinematic fibril rearrangement and fluid diffusion at given loading rates and thus delayed rupture of a collagen network and crack nucleation. For both intact and GAG-depleted cartilage, crack nucleation at the fast and slow loading rates occurred in pre- and post-relaxation timescales, respectively, and total work for crack nucleation rapidly increased toward a post-relaxation timescale. These results showed that cartilage in the vicinity of the tip experienced relatively large PVE relaxation, accompanied by kinematic fibril rearrangement and fluid diffusion, at the slow loading rates, resulting in delayed crack nucleation. The dependence of rate-dependent crack morphology on the degree of relaxation before failure provided further evidence for relaxation-governed rate-dependent crack nucleation in intact and GAG-depleted cartilage. These findings from pre- to post-relaxation timescales underlined the importance of PVE relaxation mechanisms in rate-dependent cartilage failure and provided new insight into the onset of load-induced cartilage damage. Also, these findings can be useful information for designing cartilage-like tough and dissipative hydrogel materials across a wide range of loading rates.

## Methods

### Sample preparation

Patellae of 20 porcine joints were harvested from a local abattoir (14 animals, 5–6 months old, sex unknown and assumed random) to prepare 100 full-thickness cartilage samples (Fig. 2A). Samples were randomized across the ten test conditions such that no two samples in a given test condition were from the same animal. Six mm diameter cylindrical cores were obtained using a biopsy punch and a scalpel. A bottom surface parallel to an articular surface was created by removing subchondral bone with a microtome, allowing an indenter to be placed perpendicular to the articular surface. Half of the samples underwent GAG depletion prior to testing. GAG depletion was induced by incubating cartilage in 0.25% trypsin (Corning, Manassas, VA) at 37 °C for 7 h, resulting in selective digestion of GAG without significantly affecting a collagen matrix^62,63^. GAG contents before and after the trypsin treatment were confirmed with pilot cartilage samples from the same abattoir via the dimethyl-methylene blue assay (intact cartilage: 30.55 ± 2.77 μg/mg wet weight and GAG-depleted cartilage: 2.84 ± 1.35 μg/mg wet weight)^24^. The bottom surface of each sample was fixed to the bottom of a custom well using cyanoacrylate (Loctite 495, Henkel, Germany). Dulbecco's phosphate-buffered saline (DPBS) with protease inhibitor (PI; Pierce Protease Inhibitor Mini Tablets, EDTA Free, Thermo Scientific, Waltham, MA) was used to keep samples hydrated during preparation and testing.

### Microindentation-induced crack nucleation across a broad range of loading rates

Crack nucleation in intact and GAG-depleted cartilage was induced by performing microindentation tests at multiple loading rates (Fig. 2A). Tests were performed on a 3230-AT Series III test instrument (TA Instruments, New Castle, DE) with a diamond sphero-conical indenter (tip radius: R = 100 µm and tip angle: $\uptheta$ =  90°). Under displacement control, the articular surface of intact and GAG-depleted cartilage was indented at loading rates of 5, 0.5, 0.05, and 0.005 mm s^−1^ while the load response was recorded. The sampling rates at loading rates of 5, 0.5, and 0.05 mm s^−1^ were 416.67 Hz (every 0.0024 s), and the sampling rate at a loading rate of 0.005 mm s^−1^ was 50 Hz (every 0.02 s). The broad loading rates were selected to induce crack nucleation from pre- to post-relaxation timescales of intact and GAG-depleted cartilage. Rupture of intact and GAG-depleted cartilage was identified as a sudden drop in the measured load response (Fig. 2B). In this study, the first detectable load drop with the current instrument was defined as major crack nucleation. The indentation displacement was prescribed based on preliminary data so that only a single major crack was nucleated for all of the cases except for GAG-depleted cartilage at 5 mm s^−1^. Maintaining single crack nucleation in GAG-depleted cartilage at 5 mm s^−1^ failed because sequential crack events were too close to the major crack event. Crack nucleation was quantified with critical load, *L*_*C*_, displacement, *D*_*C*_, and total work, *W*_*C*_, and critical time, *T*_*C*_ (Fig. 2). The critical total work was obtained from the integral of load–displacement curves from zero to the critical displacement (trapezoidal integration with Origin 2018 (OriginLab, Northampton, MA)). The critical time was defined as the time required for crack nucleation and was determined by dividing the critical displacement by the corresponding loading rate. Ten tests for each loading rate were conducted at the center of 10 samples.

### Relaxation responses of cartilage

Relaxation responses of intact and GAG-depleted cartilage were measured by conducting indentation tests on the articular surface. Tests were performed on the instrument with the sphero-conical indenter used for the crack nucleation tests. The indenter was moved down to a displacement of 0.3 mm (~ 18% of the average sample thickness) at a loading rate of 0.1 mm s^−1^ and then held for 200 s while the load response was measured. GAG-depleted cartilage was prepared in the same way as that for the crack nucleation tests. A total of 10 tests for each matrix integrity were performed at the center of 10 samples. Times required to reach 50, 70, and 90% of total relaxation were calculated from the results of the relaxation responses. The total relaxation was defined from maximum load to equilibrium load while the displacement was held constant. The equilibrium load was determined at 200 s of relaxation, beyond which load relaxation rates became negligibly small (~ 10^−4^ N s^−1^) compared to the initial rates (~ 1.5 N s^−1^ for intact cartilage and ~ 0.6 N s^−1^ for GAG-depleted cartilage). Unrelaxed and relaxed elastic moduli of intact and GAG-depleted tissues were calculated at the maximum (0 s of relaxation) and equilibrium (200 s of relaxation) loads through a linear elastic solution for a rigid cone indenter^64^. Pre- and post-relaxation regimes (or timescales) were divided based on the time required to reach 50% of total relaxation.

### Brightfield images of cracks

Cracks induced by microindentation tests were assessed through brightfield images. India ink was dropped on the articular surface and gently cleaned with DPBS and a delicate task wiper (Kimtech, Orange, TX). Then the articular surface was imaged using a IX-71 inverted microscope at 4 × (Olympus, Tokyo, Japan). Crack lengths and number of crack branches were measured manually using the segmented line tool in ImageJ (version 1.52a, National Institutes of Health). Each crack was measured three times and averaged to obtain the final crack length.

### Statistical analysis

The Kruskal–Wallis test was used to determine the dependence of crack nucleation results (critical load, critical displacement, critical total work, critical time, crack lengths, and the number of branches) on loading rates, and relaxation time on relaxation degree. The Mann–Whitney U test was used to statistically compare experimental results (critical load, critical displacement, critical total work, critical time, relaxation times, and elastic moduli) from intact and GAG-depleted cartilage. Non-parametric tests were used as the number of samples was small. All statistical analysis was performed using MATLAB (The MathWorks, Inc., Natick, MA). A significance level of 5% was used for all tests.

## Supplementary information

Supplementary Informations.

## Data Availability

Data are available upon reasonable request from the corresponding author.

## References

[1] Mow VC, Ratcliffe A, Robin Poole A (1992). Cartilage and diarthrodial joints as paradigms for hierarchical materials and structures. Biomaterials.

[2] Han E, Chen SS, Klisch SM, Sah RL (2011). Contribution of proteoglycan osmotic swelling pressure to the compressive properties of articular cartilage. Biophys. J..

[3] Kempson GE, Freeman MAR, Swanson SAV (1968). Tensile properties of articular cartilage. Nature.

[4] Soulhat J, Buschmann MD, Shirazi-Adl A (1999). A fibril-network-reinforced biphasic model of cartilage in unconfined compression. J. Biomech. Eng..

[5] Broom ND, Silyn-Roberts H (1990). Collagen-collagen versus collagen-proteoglycan interactions in the determination of cartilage strength. Arthritis Rheum..

[6] Schmidt MB, Mow VC, Chun LE, Eyre DR (1990). Effects of proteoglycan extraction on the tensile behavior of articular cartilage. J. Orthop. Res. Off. Publ. Orthop. Res. Soc..

[7] The US Burden of Disease Collaborators (2018). The State of US Health, 1990–2016: burden of diseases, injuries, and risk factors among US States. JAMA.

[8] Vos T (2017). Global, regional, and national incidence, prevalence, and years lived with disability for 328 diseases and injuries for 195 countries, 1990–2016: a systematic analysis for the Global Burden of Disease Study 2016. The Lancet.

[9] Wallace IJ (2017). Knee osteoarthritis has doubled in prevalence since the mid-20th century. Proc. Natl. Acad. Sci..

[10] Lawrence RC (2008). Estimates of the prevalence of arthritis and other rheumatic conditions in the United States: part II. Arthritis Rheum..

[11] Wluka AE, Lombard CB, Cicuttini FM (2013). Tackling obesity in knee osteoarthritis. Nat. Rev. Rheumatol..

[12] Rossignol M (2005). Primary osteoarthritis of hip, knee, and hand in relation to occupational exposure. Occup. Environ. Med..

[13] Buckwalter JA, Lane NE (1997). Athletics and osteoarthritis. Am. J. Sports Med..

[14] Martin, J. A., Coleman, M. & Buckwalter, J. A. Chapter 52—Articular cartilage injury, in *Principles of Tissue Engineering (Fifth Edition)* (eds. Lanza, R., Langer, R., Vacanti, J. P. & Atala, A.) 967–977 (Academic Press, 2020).

[15] Kujala UM, Kaprio J, Sarno S (1994). Osteoarthritis of weight bearing joints of lower limbs in former elite male athletes. BMJ.

[16] Mora JC, Przkora R, Cruz-Almeida Y (2018). Knee osteoarthritis: pathophysiology and current treatment modalities. J. Pain Res..

[17] Robinson WH (2016). Low-grade inflammation as a key mediator of the pathogenesis of osteoarthritis. Nat. Rev. Rheumatol..

[18] Lakes PR (2009). Viscoelastic Materials.

[19] Nia H, Han L, Li Y, Ortiz C, Grodzinsky A (2011). Poroelasticity of cartilage at the nanoscale. Biophys. J..

[20] Mow VC, Kuei SC, Lai WM, Armstrong CG (1980). Biphasic creep and stress relaxation of articular cartilage in compression: theory and experiments. J. Biomech. Eng..

[21] Huang C-Y, Soltz MA, Kopacz M, Mow VC, Ateshian GA (2003). Experimental verification of the roles of intrinsic matrix viscoelasticity and tension-compression nonlinearity in the biphasic response of cartilage. J. Biomech. Eng..

[22] Mak AF (1986). The apparent viscoelastic behavior of articular cartilage—The contributions from the intrinsic matrix viscoelasticity and interstitial fluid flows. J. Biomech. Eng..

[23] Han G, Hess C, Eriten M, Henak CR (2018). Uncoupled poroelastic and intrinsic viscoelastic dissipation in cartilage. J. Mech. Behav. Biomed. Mater..

[24] Han G, Boz U, Eriten M, Henak CR (2020). Glycosaminoglycan depletion increases energy dissipation in articular cartilage under high-frequency loading. J. Mech. Behav. Biomed. Mater..

[25] Wahlquist JA (2017). Indentation mapping revealed poroelastic, but not viscoelastic, properties spanning native zonal articular cartilage. Acta Biomater..

[26] Ateshian GA, Warden WH, Kim JJ, Grelsamer RP, Mow VC (1997). Finite deformation biphasic material properties of bovine articular cartilage from confined compression experiments. J. Biomech..

[27] Lawless BM (2017). Viscoelasticity of articular cartilage: Analysing the effect of induced stress and the restraint of bone in a dynamic environment. J. Mech. Behav. Biomed. Mater..

[28] Nia H (2013). High-bandwidth AFM-based rheology reveals that cartilage is most sensitive to high loading rates at early stages of impairment. Biophys. J..

[29] Sadeghi H, Lawless BM, Espino DM, Shepherd DET (2018). Effect of frequency on crack growth in articular cartilage. J. Mech. Behav. Biomed. Mater..

[30] Kaplan JT, Neu CP, Drissi H, Emery NC, Pierce DM (2017). Cyclic loading of human articular cartilage: the transition from compaction to fatigue. J. Mech. Behav. Biomed. Mater..

[31] Sadeghi H, Shepherd DET, Espino DM (2015). Effect of the variation of loading frequency on surface failure of bovine articular cartilage. Osteoarthr. Cartil..

[32] Kaleem B, Maier F, Drissi H, Pierce DM (2017). Low-energy impact of human cartilage: predictors for microcracking the network of collagen. Osteoarthr. Cartil..

[33] Su AW (2018). Biomechanics of osteochondral impact with cushioning and graft Insertion: cartilage damage is correlated with delivered energy. J. Biomech..

[34] Henak CR, Bartell LR, Cohen I, Bonassar LJ (2017). Multiscale strain as a predictor of impact-induced fissuring in articular cartilage. J. Biomech. Eng..

[35] Bartell LR, Xu MC, Bonassar LJ, Cohen I (2018). Local and global measurements show that damage initiation in articular cartilage is inhibited by the surface layer and has significant rate dependence. J. Biomech..

[36] Han G, Eriten M, Henak CR (2019). Rate-dependent crack nucleation in cartilage under microindentation. J. Mech. Behav. Biomed. Mater..

[37] Buckwalter JA (1992). Mechanical injuries of articular cartilage. Iowa Orthop. J..

[38] Simon TM, Jackson DW (2018). Articular cartilage: injury pathways and treatment options. Sports Med. Arthrosc. Rev..

[39] Guilak F, Ratcliffe A, Lane N, Rosenwasser MP, Mow VC (1994). Mechanical and biochemical changes in the superficial zone of articular cartilage in canine experimental osteoarthritis. J. Orthop. Res..

[40] Sui Y (2009). Mechanical injury potentiates proteoglycan catabolism induced by interleukin-6 with soluble interleukin-6 receptor and tumor necrosis factor α in immature bovine and adult human articular cartilage. Arthritis Rheum..

[41] Simha NK, Carlson CS, Lewis JL (2004). Evaluation of fracture toughness of cartilage by micropenetration. J. Mater. Sci. Mater. Med..

[42] Han L (2011). Time-dependent nanomechanics of cartilage. Biophys. J..

[43] Bouklas N, Landis CM, Huang R (2015). A nonlinear, transient finite element method for coupled solvent diffusion and large deformation of hydrogels. J. Mech. Phys. Solids.

[44] Hu Y, Zhao X, Vlassak JJ, Suo Z (2010). Using indentation to characterize the poroelasticity of gels. Appl. Phys. Lett..

[45] Zhu W, Iatridis JC, Hlibczuk V, Ratcliffe A, Mow VC (1996). Determination of collagen-proteoglycan interactions in vitro. J. Biomech..

[46] Shergold OA, Fleck NA (2005). Experimental investigation into the deep penetration of soft solids by sharp and blunt punches, with application to the piercing of skin. J. Biomech. Eng..

[47] Shergold OA, Fleck NA (2004). Mechanisms of deep penetration of soft solids, with application to the injection and wounding of skin. Proc. R. Soc. Lond. Ser. Math. Phys. Eng. Sci..

[48] Chin-Purcell MV, Lewis JL (1996). Fracture of articular cartilage. J. Biomech. Eng..

[49] Adams, D. J., Brosche, K. M. & Lewis, J. L. Factors affecting fracture parameters for articular cartilage. in *45th Annual Meeting. Orthop. Res. Soc.* 1–4 (1999).

[50] Han G, Eriten M (2018). Effect of relaxation-dependent adhesion on pre-sliding response of cartilage. R. Soc. Open Sci..

[51] Vandenberghe N, Villermaux E (2013). Geometry and fragmentation of soft brittle impacted bodies. Soft Matter.

[52] Moulinet S, Adda-Bedia M (2015). Popping balloons: a case study of dynamical fragmentation. Phys. Rev. Lett..

[53] Chen C, Wang Z, Suo Z (2017). Flaw sensitivity of highly stretchable materials. Extreme Mech. Lett..

[54] Hunziker EB, Quinn TM, Häuselmann H-J (2002). Quantitative structural organization of normal adult human articular cartilage. Osteoarthr. Cartil..

[55] Ng L (2003). Individual cartilage aggrecan macromolecules and their constituent glycosaminoglycans visualized via atomic force microscopy. J. Struct. Biol..

[56] Roughley PJ, Lee ER (1994). Cartilage proteoglycans: structure and potential functions. Microsc. Res. Tech..

[57] Vazquez KJ, Andreae JT, Henak CR (2019). Cartilage-on-cartilage cyclic loading induces mechanical and structural damage. J. Mech. Behav. Biomed. Mater..

[58] Deneweth JM, Newman KE, Sylvia SM, McLean SG, Arruda EM (2013). Heterogeneity of tibial plateau cartilage in response to a physiological compressive strain rate. J. Orthop. Res..

[59] Liu F (2010). In vivo tibiofemoral cartilage deformation during the stance phase of gait. J. Biomech..

[60] Hosseini A (2010). In-vivo time-dependent articular cartilage contact behavior of the tibiofemoral joint. Osteoarthritis Cartilage.

[61] June RK, Fyhrie DP (2010). Temperature effects in articular cartilage biomechanics. J. Exp. Biol..

[62] Griffin DJ (2014). Effects of enzymatic treatments on the depth-dependent viscoelastic shear properties of articular cartilage. J. Orthop. Res..

[63] Nguyen Q, Murphy G, Roughley PJ, Mort JS (1989). Degradation of proteoglycan aggregate by a cartilage metalloproteinase. Evidence for the involvement of stromelysin in the generation of link protein heterogeneity in situ. Biochem. J..

[64] Briscoe BJ, Sebastian KS, Adams MJ (1994). The effect of indenter geometry on the elastic response to indentation. J. Phys. Appl. Phys..

[65] Li J, Illeperuma WRK, Suo Z, Vlassak JJ (2014). Hybrid hydrogels with extremely high stiffness and toughness. ACS Macro Lett..

[66] Yang CH (2013). Strengthening alginate/polyacrylamide hydrogels using various multivalent cations. ACS Appl. Mater. Interfaces.

[67] Sun J-Y (2012). Highly stretchable and tough hydrogels. Nature.

[68] Rivlin RS, Thomas AG (1953). Rupture of rubber. I. Characteristic energy for tearing. J. Polym. Sci..

[69] Wegst UGK, Ashby MF (2004). The mechanical efficiency of natural materials. Philos. Mag..

