# Supplementary material for: Relaxation capacity of cartilage is a critical factor in rate- and integrity-dependent fracture
Source: Sci Rep. 2021 May 4;11:9527. doi: 10.1038/s41598-021-88942-w (PMC8096812; doi:10.1038/s41598-021-88942-w)
Supplement: Supplementary file 1 — Supplementary Informations. [file 41598_2021_88942_MOESM1_ESM.docx]

Supplementary Information for

Relaxation capacity of cartilage is a critical factor in rate- and integrity-dependent fracture

G. Han, U. Chowdhury, M. Eriten, and C. R. Henak*

*Corresponding author. Email: [chenak@wisc.edu](mailto:chenak@wisc.edu)

**The file includes:**

Supplementary Text

Supplementary Figures (Figs. S1 to S4)

**Supplementary Text**

**Determination of the critical energy release rate of cartilage**

The consistent line-shaped cracks in intact (5 and 0.5 mm∙s^-1^) and GAG-depleted (5 mm∙s^-1^) cartilage, generated within a pre-relaxation timescale (Fig. 4A and C), allowed us to determine the critical energy release rates by using a penetration model for a sharp punch proposed by Shergold and Fleck ^1,2^.

In preparation for calculating the critical energy release rates of intact and GAG-depleted cartilage, their shear moduli and contact radii just prior to crack nucleation were estimated by analyzing the experimental results with an axisymmetric finite element (FE) model (Fig. S3A). All of the modeling and simulation were performed in Abaqus 2018 (Dassault Systemes SIMULIA, RI, USA). The sphero-conical indenter model had dimensions identical to the experimental setup (tip radius: 100 µm and half-angle of cone: 45º). The cartilage model dimensions were 3 mm in radius and 1.6 mm in thickness based on the measurement of intact cartilage samples (radius: 3 mm and thickness: 1.56 ± 0.11 mm). The bottom surface of the cartilage model was fixed in all directions. Contact between the indenter and cartilage models was set as frictionless. Surface to surface interaction was prescribed at the interface, and the penalty method was used to enforce the contact constraints. The indenter and cartilage models were considered as the master and slave surfaces, respectively. The cartilage model was discretized through biased meshing, resulting in finer meshes toward the contact area. Four quarter circles with radii of 0.25 mm, 0.5 mm, 0.75 mm, and 1 mm were generated. The minimum and maximum mesh sizes of 0.001 and 0.005 mm were assigned to the smallest and largest quarter circles, respectively. The mesh size outside the largest quarter circle was set to be 0.03 mm. The model was composed of quadrilateral (CAX4R: 98419 elements) and triangular (CAX3: 2338 elements) elements. Linear interpolation between nodes was used. The number of elements was determined through a mesh convergence analysis.

The indenter and cartilage were modeled as a rigid body and a hyperelastic Ogden material, respectively. The Ogden material model was selected to match the penetration model for a sharp punch ^1,2^. Since crack nucleation in intact (5 and 0.5 mm∙s^-1^) and GAG-depleted (5 mm∙s^-1^) cartilage occurred in a pre-relaxation timescale (Fig. 4A), it was reasonable to assume that cartilage behaved as a nearly-incompressible nonlinear elastic solid. The strain energy density of the Ogden material model is expressed by

|  | $U=\sum_{i=1}^{N} \frac{2\mu_{i}}{\alpha_{i}^{2}}(\lambda_{1}^{\alpha_{i}}+\lambda_{2}^{\alpha_{i}}+\lambda_{3}^{\alpha_{i}}-3)$ | (1) |
| --- | --- | --- |

Where $\mu_{i}$ is the shear modulus, $\lambda_{1,2,and3}^{i}$are the deviatoric principal stretches, and $\alpha_{i}$ is the power law constant. $N$was set as 1. $\mu_{1}$ and $\alpha_{1}$ were set as fitting parameters. The small-strain bulk modulus, $K$, was given by

|  | $K=\frac{2\mu_{1}(1+v)}{3(1-2v)}$ | (2) |
| --- | --- | --- |

where $v$ is Poisson’s ratio. Since cartilage in a pre-relaxation timescale behaved as a nearly incompressible material, $v$ was set to 0.5. $\mu_{1}$ was determined by manually fitting the FE-predicted load-displacement curves to the average experimental load-displacement curves of intact (5 and 0.5 mm∙s^-1^) and GAG-depleted (5 mm∙s^-1^) cartilage (R^2^ > 0.97) (Fig. S3B). As a result, $\mu_{1}$ for intact cartilage was determined to be 10 MPa (5 mm∙s^-1^) and 8 MPa (0.5 mm∙s^-1^), respectively. $\mu_{1}$ for GAG-depleted cartilage was determined to be 6 MPa (5 mm∙s^-1^). In addition, a contact radius as a function of displacement (Fig. S3C) was obtained to determine the contact radii, $R_{c}$, corresponding to critical displacements (Fig. 2C). $R_{c}$ was calculated from a linear function fitted to the FE-predicted contract radius curve (R^2^ > 0.99).

The penetration model for a sharp punch ^1,2^ provided a master curve for the critical energy release rate of a hyperplastic solid. In the penetration model, a rigid conical tip with a cylindrical shaft of radius, $R$, was indented into a semi-infinite solid. The solid was considered as an incompressible, hyperelastic, and isotropic material and modeled with the Ogden material model. The contact between the tip and solid was considered to be frictionless. The steady-state penetration of the tip by an axial increment, $\delta l$, was caused due to a load, $P_{S}$, while generating a plane strain crack with a length of $2a$ and a thickness of $\delta l$ and then opening the crack to accommodate the tip. The energy balance approach was used by equating the work done by the penetrating indenter tip, $P_{S}$ $\delta l$, to the sum of the energy required to extend a crack, $\delta W_{C}$, and the strain energy increase for crack surface to accommodate the penetrating indenter, $\delta S_{E}$, as follows:

| $P_{S}\delta l=\delta W_{C}+\delta S_{E}$. | (3) |
| --- | --- |

When a crack with a length of $2a$ propagates to a depth of $\delta l$, $\delta W_{C}$was equal to $2J_{IC}a\delta l$for the mode-I toughness of the material, $J_{IC}$. $\delta S_{E}$ was estimated via the FE simulation and expressed in terms of a dimensionless function, $h\left(\frac{a}{R}\right)$, for the convenient FE analysis. Then, Eq. 3 is given by

| $P_{s}\delta l=2J_{IC}a\delta l+\mu R^{2}h\left( \frac{a}{R} \right)\delta l$. | (4) |
| --- | --- |

The average penetration pressure on the punch, $p_{S}$, is obtained by dividing $P_{S}$ by the cross section ($=\pi R^{2}$). As a result, Eq. 4 is expressed by

| $\frac{p_{s}}{\mu}=\frac{P_{s}}{\pi\mu R^{2}}=\frac{2}{\pi}\left( \frac{J_{1C}}{\mu R} \right)\left( \frac{a}{R} \right)+\frac{1}{\pi}h\left( \frac{a}{R} \right)$. | (5) |
| --- | --- |

When $\frac{p_{S}}{\mu}$ is plotted as a function of $\frac{a}{R}$ (Eq. 5), the stable penetration occurs at the minimum value of $\frac{p_{S}}{\mu}$ (= $\frac{p_{S-\min}}{\mu}$). Shergold and Fleck ^2^ predicted the relationship between $\frac{p_{S-\min}}{\mu}$, $\frac{a}{R}$, $\frac{J_{IC}}{\mu R}$, and $\alpha$ through the FE analysis. The master curve providing the relationship between $\frac{a}{R}$ and $\frac{p_{S-\min}}{\mu}$ for $\alpha_{1}$ = 5 ^2^ was replotted in Fig. S3D to determine the critical energy release rates of intact and GAG-depleted cartilage. In our study, $R$ was equal to $R_{c}$ which was obtained via the FE simulation based on the experimental setup and results (Fig. S3C and Fig. 2C).

The critical energy release rates of intact and GAG-depleted cartilage were determined by projecting the experimental and FE-predicted values on the master curve showing the relationship between $\frac{a}{R}$ and $\frac{p_{S-\min}}{\mu}$ from the penetration model for a sharp punch ^1,2^ (Fig. S3D). The experimentally measured values of critical load (Fig. 2C) and crack lengths (Fig. 4B) for intact and GAG-depleted cartilage provided the values of $P_{S}$ and $a$, respectively; $p_{S-\min}$ was calculated through $\frac{P_{S}}{\pi R_{c}^{2}}$. $R$ and $\mu$ were obtained from the FE simulation ($R_{c}$ and $\mu_{1}$) based on the experimental results, described in the previous paragraphs. Consequently, the $\frac{a}{R}$ and $\frac{p_{S-\min}}{\mu}$ determined for intact (5 and 0.5 mm∙s^-1^) and GAG-depleted (5 mm∙s^-1^) cartilage formed clusters close to the master curve for $\alpha_{1}$ = 5 ^2^ (Fig. S3D). It was determined to use the master curve for $\alpha_{1}$ = 5 because it was placed close to the experimental data points. For example, the average distance between the master curve for $\alpha_{1}$ = 5 (0.10$\pm$0.10) and the three points was shorter than the distances with the master curves for $\alpha_{1}$ = 3 (0.97$\pm$0.10) and $\alpha_{1}$ = 9 (0.29$\pm$0.11). This showed that the penetration model for a sharp punch ^1,2^ can be used to determine the critical energy release rates of intact and GAG-depleted cartilage in pre-relaxation timescales. This reiterated that intact and GAG-depleted cartilage did not have enough time to relax at fast loading rates. $\frac{J_{IC}}{\mu R}$ corresponding to $\frac{a}{R}$ determined in intact cartilage (1.23 ± 0.21 for 5 mm∙s^-1^ and 1.21 ± 0.13 for 0.5 mm∙s^-1^) was 1.34 ± 0.74 for 5 mm∙s^-1^ and 1.25 ± 0.58 for 0.5 mm∙s^-1^ ^2^. $\frac{J_{IC}}{\mu R}$ corresponding to $\frac{a}{R}$ determined in GAG-depleted cartilage (1.39 ± 0.31 for 5 mm∙s^-1^) was 1.31 ± 1.85 for 5 mm∙s^-1^ ^2^. Since $\mu$ and $R$ were already obtained from the FE simulation, the critical energy release rates of intact cartilage were determined as 2.39 ± 1.39 kJ∙m^-2^ for 5 mm∙s^-1^ and 2.48 ± 1.26 kJ∙m^-2^ for 0.5 mm∙s^-1^. The critical energy release rate of GAG-depleted cartilage was determined as 2.09 ± 2.99 kJ∙m^-2^ for 5 mm∙s^-1^; this value could be overestimated because one specific data point was high (10.43 kJ∙m^-2^); the average value without the data point was 1.17 ± 0.62 kJ∙m^-2^. The estimated critical energy release rates were valid under the assumption that minute failure in cartilage, not sufficiently large to be captured by the instrument, was induced at the end of the indenter tip and was led to a detectable failure event. This failure was detected by a sudden drop in the measured load response and was defined as crack nucleation in this current study. Possible sources of error could originate from the assumptions of the penetration model such as the stable crack and the frictionless interactions at the contact ^2^. The crack lengths of GAG-depleted cartilage in a pre-relaxation timescale (5 mm∙s^-1^) might also have been overestimated due to small crack events after major crack nucleation events, suggested from the fluctuations in the slopes of the load-displacement curves; on the other hand, single major crack nucleation events only occurred in other sample and loading conditions.

**Supplementary figures**


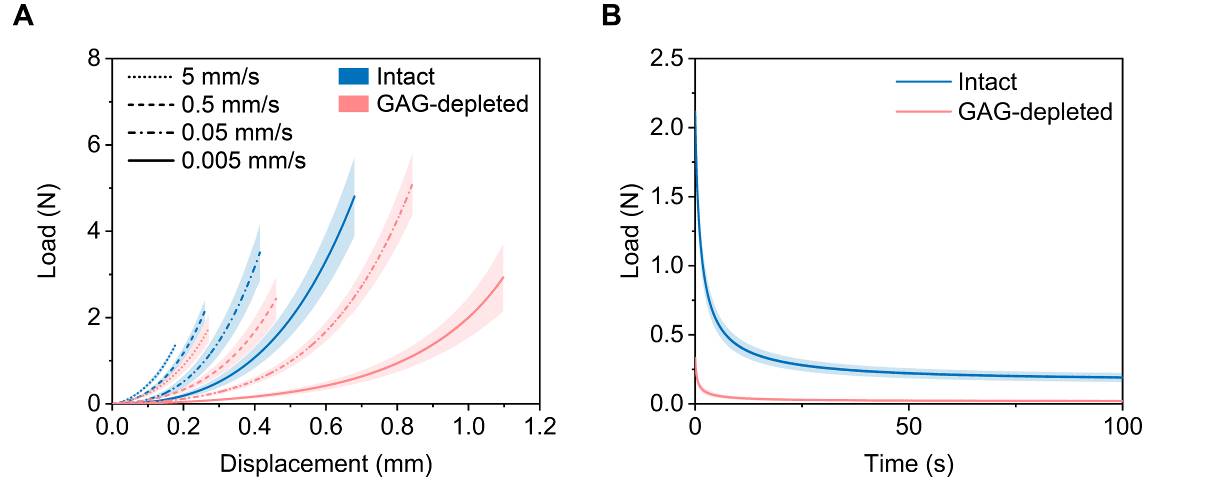


Fig. S1. Experimental results of crack nucleation and relaxation tests. (A) Average load-displacement curves prior to crack nucleation. The average load-displacement curves were obtained up to the minimum critical displacements at corresponding loading rates. However, the average curve for GAG-depleted cartilage at 0.005 mm∙s^-1^ was up to the end of a prescribed displacement because crack nucleation did not occur. The prescribed displacement was the maximum displacement limit determined to prevent the diamond microindenter from potential damage. (B) Average relaxation curves.


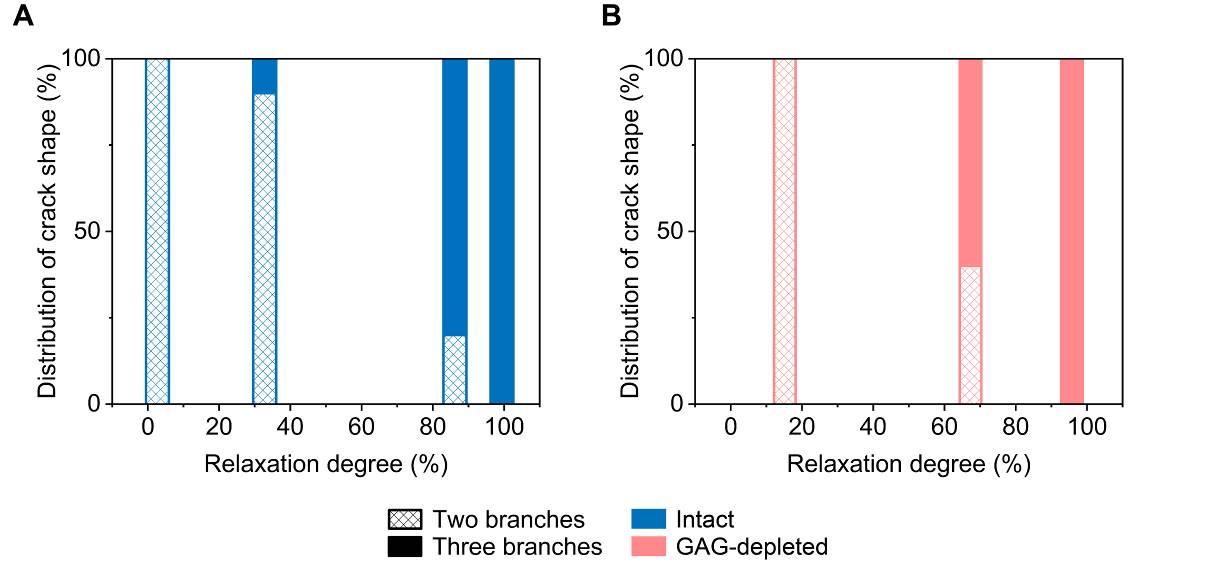


Fig. S2. Number of crack branches. (A) Intact cartilage. (B) GAG-depleted cartilage. The number of branches for a line-shaped crack was counted as two branches extended from the center of the crack. The number of branches increased from two to three with an increase in relaxation degree.


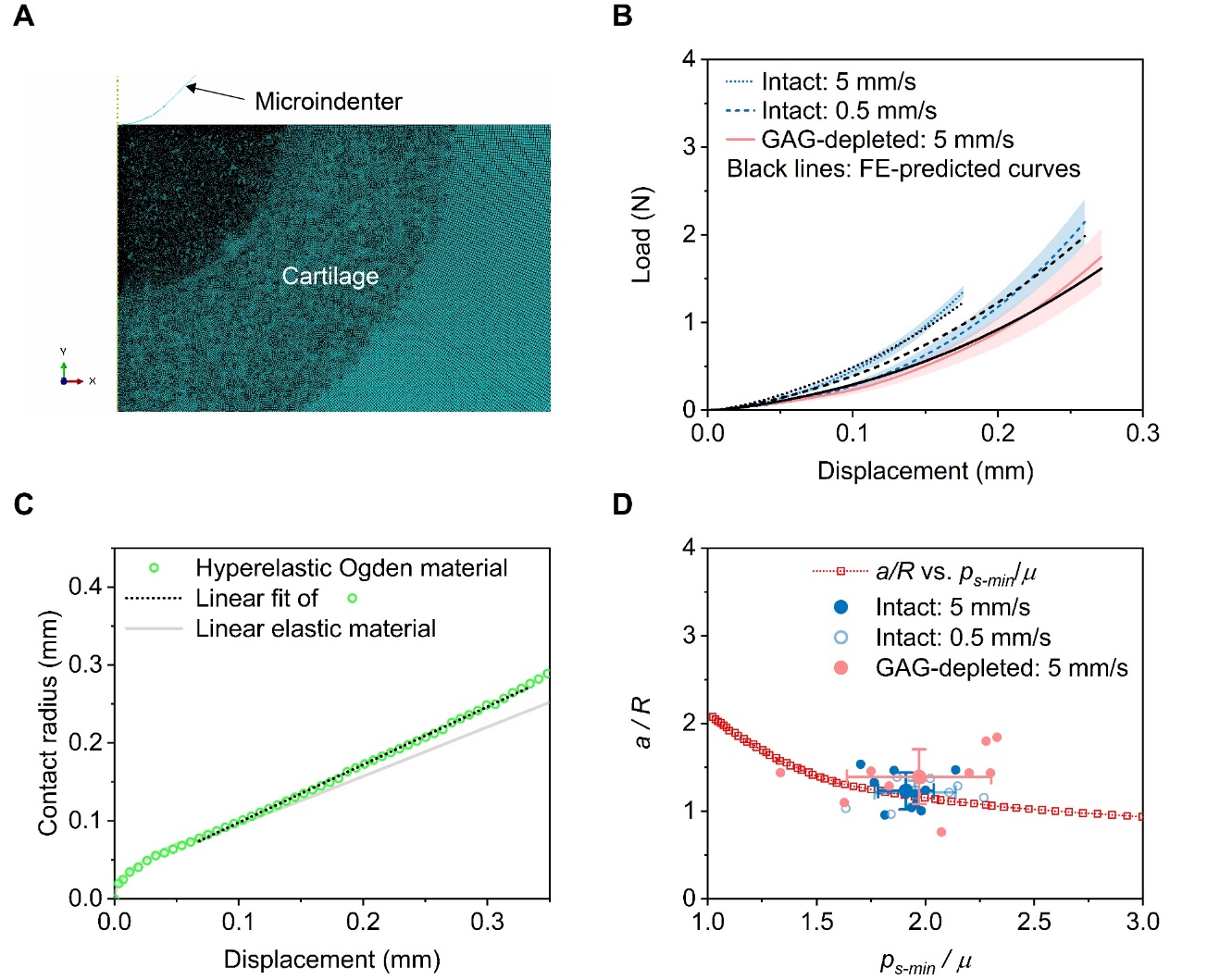


Fig. S3. Estimation of the critical energy release rates of intact and GAG-depleted cartilage in a pre-relaxation timescale. (A) FE model of an indenter-cartilage system. The mesh size became finer toward the contact area between the indenter and cartilage models. (B) Comparison between experimental and FE-predicted load-displacement curves (R^2^ > 0.97 for all curves). (C) Contact radii as a function of a displacement. The contact radius estimated from the Ogden material was slightly larger than that previously estimated from a linear elastic material ^3^. A linear function was fitted to the FE-predicted curve (R^2^ > 0.99). (D) Master curve for the relationship between $\frac{a}{R}$ and $\frac{\mathbf{p}_{\mathbf{s-min}}}{\boldsymbol{\mu}}$ with projected data points. The master curve was taken from a previous study ^2^. The projected data points were determined using the experimental and simulated results.

**
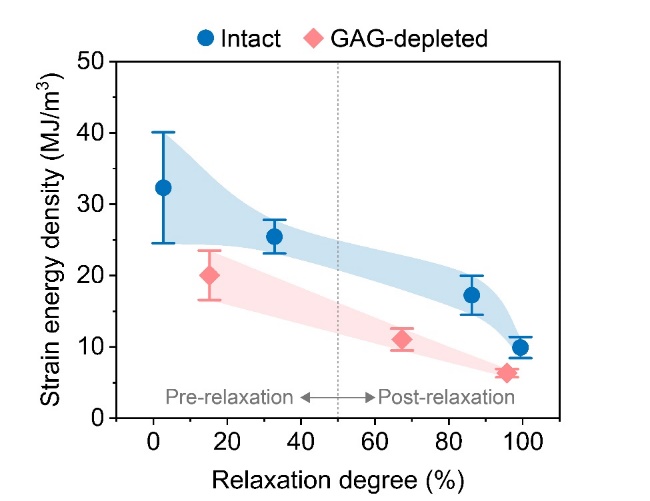
**

**Fig. S4. Estimation of the strain energy density as functions of relaxation degree. Strain energy density for the indentation of an elastic half space can be estimated by dividing mechanical total work by a highly stressed volume in the vicinity of an indenter. The volume can be approximated with the cube of a contact radius obtained with a linear elastic solution for a sphero-conical indenter in Fig. S3C. Using a contact radius as a characteristic length in the system is reasonable as high stress fields are concentrated within a contact radius** ^4^**. When mechanical total work reaches critical total work (Fig. 2), strain energy density was considered as work to fracture (Fig. 5) having the dimension of energy per unit volume.**

**References**

1. Shergold, O. A. & Fleck, N. A. Experimental Investigation Into the Deep Penetration of Soft Solids by Sharp and Blunt Punches, With Application to the Piercing of Skin. *J. Biomech. Eng.* **127**, 838–848 (2005).

2. Shergold, O. A. & Fleck, N. A. Mechanisms of deep penetration of soft solids, with application to the injection and wounding of skin. *Proc. R. Soc. Lond. Ser. Math. Phys. Eng. Sci.* **460**, 3037–3058 (2004).

3. Briscoe, B. J., Sebastian, K. S. & Adams, M. J. The effect of indenter geometry on the elastic response to indentation. *J. Phys. Appl. Phys.* **27**, 1156 (1994).

4. Sneddon, I. N. Boussinesq’s problem for a rigid cone. *Math. Proc. Camb. Philos. Soc.* **44**, 492–507 (1948).
